# In silico exploring of the epigenetic factors in teratozoospermia: A focus on *IGF2BP2*

**DOI:** 10.22099/mbrc.2025.52777.2123

**Published:** 2025

**Authors:** Seyedeh Zahra Mousavi, Bahram Mohammad-Soltani, Morteza Hadizadeh, Zeynab Rokhsattalab, Mehdi Totonchi

**Affiliations:** 1 Department of Molecular Genetics, Faculty of Biological Sciences, Tarbiat Modares University, Tehran, Iran; 2 Physiology Research Center, Institute of Neuropharmacology, Kerman University of Medical Sciences, Kerman, Iran; 3 Department of Genetics, Reproductive Biomedicine Research Center, Royan Institute for Reproductive Biomedicine, ACECR, Tehran, Iran

**Keywords:** Epigenetic markers, Teratozoospermia, RNA-protein interaction, RNA-binding proteins, IGF2BP2

## Abstract

Teratozoospermia is an abnormal sperm morphology that is a common cause of male infertility. Epigenetic factors have been implicated in the regulation of gene expression in teratozoospermia, but the specific mechanisms are not fully understood. This study aimed to identify differentially expressed genes (DEGs) between teratozoospermia and normozoospermia samples, and to investigate the role of epigenetic regulatory factors in the observed gene expression changes. The study integrated data from three publicly available datasets in the GSE6969 superseries. The DEGs were compared to a list of known human epigenetic-related genes obtained from the EpiFactors database. The protein-protein interaction (PPI) network and hub gene identifications for Epi-DEGs and the RNA-protein interaction (RPI) network to obtained the RBPs interacting with Epi-DEGs were constructed. siRNA design for the candidate mRNA was performed using various bioinformatics tools. As a result, the obtained 1,292 DEGs were compared to a list of 796 known human epigenetic factors, revealing 63 Epi-DEGs. The PPI network of Epi-DEGs identified top 10 hub genes including *RBBP7*, *HDAC2*, *EZH2*, *SMARCA5*, *SUV39H1*, *CTCF*, *DNMT1*, *MORF4L1*, *ARID4B* and *KDM5B*. The RPI network analysis revealed *IGF2BP2*, *SFPQ* and *A1CF* as key RNA-binding protein regulators epigenetic modifiers. Based on these findings, the study designed the sequence GCAACAAGAGAAGAA GCAATT as an optimal siRNA candidate targeting the master regulator IGF2BP2, which exhibited the most significant change in expression among the RNA-binding proteins (RBPs). This integrative analysis sheds light on the epigenetic mechanisms underlying teratozoospermia and highlights the potential of RBPs as diagnostic biomarkers and therapeutic targets for further investigation.

## INTRODUCTION

Infertility is a condition characterized by the inability to conceive after one year of regular, unprotected sexual intercourse [1]. In recent years, male infertility has become a major global public health concern with complex etiology [2]. Teratozoospermia refers to a range of conditions characterized by abnormal sperm phenotypes, which can affect the sperm head, neck, and tail, or a combination of these parts [3]. Epigenetics provides valuable insights into the complex origins of these diverse disorders, offering a deeper understanding of their development. The development and differentiation of the cells rely on epigenetic changes [4]. Emerging research suggests an association between aberrant sperm epigenetic modifications and altered spermatogenesis [5, 6]. Recent studies have demonstrated that epigenetic modifications can affect the expression profile in male reproductive system during development and sperm production. This indicates that dis-regulation of epigenetic modifications can lead to abnormal male sexual development and reproductive dysfunction. Epigenetic mechanisms not only control spermatogenesis, but also modulate sperm maturation, sperm-egg interaction, fertilization, and embryo development [7]. Additionally, multiple factors across various biological levels have been shown to contribute to infertility phenotypes [8]. 

Microarray technology represents a groundbreaking advancement in molecular research,  enabling the simultaneous analysis of thousands of genes and providing unprecedented insights into gene expression patterns [9]. This study is designed to identify fluctuation of epigenetic factors in teratozoospermia by integrating and re-analyzing three public and related datasets. We aim to investigate the potential role of dysregulated epigenetic factors in teratozoospermia by application of computational systems biology.

## MATERIALS AND METHODS

### Data selection and processing:

 The Gene Expression Omnibus (GEO) database was used to find relevant human teratozoospermia samples [10]. Three gene expression profiles (GSE6967, GSE6968 and GSE6872) that belonged to the GSE6969 superseries were found. These datasets utilized different microarray platforms including GPL2507 (Sentrix Human-6 Expression BeadChip), GPL2700 (Sentrix HumanRef-8 Expression BeadChip) and GPL570 ([HG-U133_Plus_2] Affymetrix Human Genome U133 Plus 2.0 Array). A list of genes involved in regulating of epigenetic processes from the EpiFactors database was selected, named EpiGenes_main [11]. Data processing and integration were performed using the R statistical programming language. After combining three GEO datasets, the data were checked for logarithmic form through minimum and maximum values. The batch effects (non-biological variations) were eliminated using the ComBat function from the R package called Surrogate Variable Analysis (SVA ([12]. Finally, a unified expression matrix was generated. Differentially expressed genes (DEGs) were extracted from the expression matrix by comparing “Teratozoospermia” and “Normozoospermia" sample groups using the R package limma [13]. A log2 fold change threshold of ≥ |1| and an adjusted p-value of < 0.01 were applied to determine statistical significance. The Venny 2.1 tool was used to intersect the HGNC_symbol column of the EpiGenes_main file extracted from the EpiFactors database (version 2.1) with the set of DEGs identified from the microarray analysis. This allowed to determine the overlap between known epigenetic factors and the genes that were DEGs between teratozoospermia and normozoospermia samples, hereafter we called Epi-DEGs).

### Gene ontology (GO) and pathway enrichment analyses:

Pathway and enrichment analyses were conducted to identify the biological mechanisms associated with the DEGs and the Epi-DEGs. The Enrichr and SRPLOT tools were utilized to perform the pathway (from Reactome 2022 database) and GO enrichment analyses (biological processes (BP), cellular components (CC), and molecular functions (MF)) on the gene list of Epi-DEGs [14-16].

### RNA-protein interaction (RPI) network analysis:

 The list of Epi-DEGs was subjected to RNAInter database, which is a comprehensive repository of RNA-associated interactions, to obtain the proteins interacting with Epi-DEGs and a human mRNA-protein interaction network [17]. The threshold score was >0.1. The obtained RPI where intersect with DEGs using the online tool Venny 2.1. and RPI network was then visualized using the Cytoscape software (v.3.10.2) [18]. 

### Protein-protein interaction (PPI) network analysis:

The list of Epi-DEs was then subjected to the STRING database (https://string-db.org/) to obtain a PPI network. The interactors where intersect with the set of epigenetic-related DEGs. The Epi-DEs were then used to construct a PPI network. Then the PPI network was visualized using the Cytoscape (3.10.2) software. 

### Hub-genes identification and validation:

 The outcome of PPI network construction was analyzed using the CytoHubba (using the Maximal Clique Centrality (MCC) function) Cytoscape plugin to identify the most central regulatory nodes (hub genes) within the PPI network. Hub genes are often considered as the central, highly connected nodes that may play critical regulatory roles. Finally, the top 10 hub genes identified by CytoHubba were selected according to the highest score. Receiver operating characteristic (ROC) analysis was performed to assess the diagnostic potential of the differentially expressed hub genes [19]. The area under the ROC curve (AUC) was calculated for the top 10 candidate hub genes to evaluate their diagnostic potential. This statistical analysis was conducted using the GraphPad Prism software (version 9.1.0) [20]. 

### siRNA design:

 The coding sequence (CDS) of the candidate mRNA transcript was obtained from the NCBI GenBank database (https://www.ncbi.nlm.nih.gov/). The rational design of the small interfering RNA (siRNA) molecules was performed using the Sfold online web server to target the candidate mRNA [21]. After designing the siRNA candidates, the CLC Genomics Workbench software was used to align the siRNA sequences against the predicted secondary structure of the candidate mRNA to detect whether siRNAs are complementary to the appropriate regions of the candidate mRNA structure or not [22]. In the next step, the designed siRNAs were evaluated using the IDT Oligoanalyzer web server to check the structural stability of the siRNAs [23]. After choosing the best-designed siRNA based on thermodynamic energy and GC content results, the binding affinity of the best siRNA with their target sites was evaluated using the HNADOCK server, and then the similarity of the best-designed siRNA sequence was compared with other genes in the human genome using the online Ensembl database to ensure that they will only bind to their particular target sequence in the candidate mRNA [24, 25].

## RESULTS

The integrated analysis of the three GEO datasets resulted in the identification of 23612 genes and 1292 DEGs between Teratozoospermia (n=22) and Normozoospermia samples (n=22) (Table S1 and S2). In total, 796 human epigenetic factors were identified from the EpiFactors database (Table S3). By comparing the DEGs with the list of epigenetic-related factors, 63 Epi-DEGs were identified. The list of the Epi-DEGs factors and their information are listed in (Table S4).

**Figure 1:**
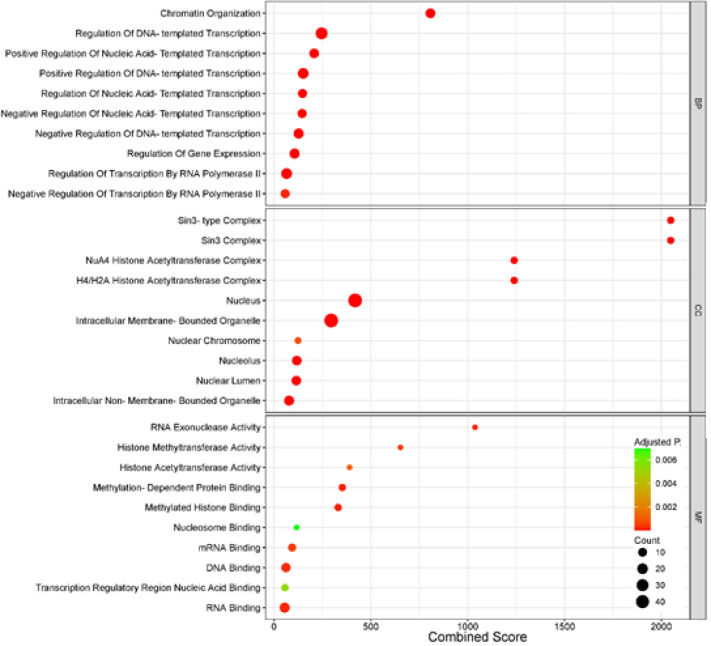
GO analysis for Epi-DEGs.

**Figure 2:**
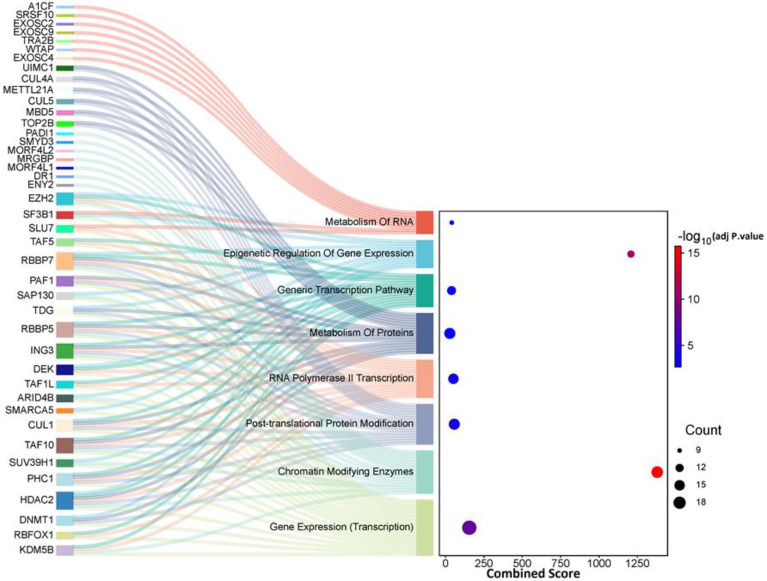
Visualizations Pathway Enrichment Analysis for Epi-DEGs.

The Enrichr database was used to identify enriched Reactome 2022 pathways with the all DEGs and Epi-DEGs, as well as GO terms for Epi-DEGs associated a *p*-adjusted value threshold of <0.01 was applied to determine statistical significance. The results of this enrichment analysis for all DEGs are shown in Table 1. The results for the Epi-DEGs are presented using two visualization formats: snacky and dot plots for the enriched Reactome 2022 pathways and bubble plots for the enriched GO terms, including BP, CC, and MF as shown in Figure 1, Figure 2, and Table S5. By integrating the pathway and ontology enrichment results, it is possible to gain a valuable comprehensive understanding of the functional roles and biological contexts associated with the Epi-DEGs.

**Table 1:** Top 10 pathway enrichment analysis for all DEGs

**Nubmer**	**Name**	**Adjusted ** ***p*** **-value**
1	Regulation of Expression of SLITs and ROBOs	6.163e-40
2	Signaling by ROBO Receptors	1.909e-34
3	Peptide Chain Elongation	1.909e-34
4	Eukaryotic Translation Elongation	2.292e-34
5	L13a-mediated Translational Silencing of Ceruloplasmin Expression	2.292e-34
6	GTP Hydrolysis and Joining of the 60S Ribosomal Subunit	3.498e-34
7	SRP-dependent Cotranslational Protein Targeting to Membrane	9.473e-34
8	Formation of a Pool of Free 40S Subunits	9.473e-34
9	Eukaryotic Translation Termination	1.095e-33
10	Nonsense Mediated Decay (NMD) Independent of the Exon Junction Complex (EJC)	3.814e-33

Bubble Plot of Top 10 GO Terms: This bubble plot displays the most significantly enriched GO terms, including Biological Processes (BP), Cellular Components (CC), and Molecular Functions (MF). The size of the bubbles represents the number of genes associated with each GO term, while the color indicates the adjusted *p*-value.

Snaky and Dot Plot of Enriched Pathways: The enriched Reactome 2022 pathways associated with the Epi-DEGs. The snaky plot presents the pathway names, while the dot plot indicates the strength of enrichment for each pathway, as measured by the adjusted *p*-value.

**Figure 3:**
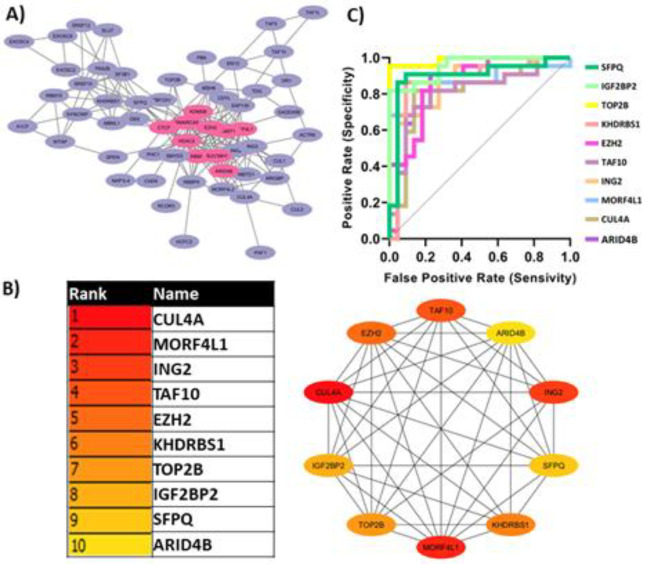
Identification and Validation of Hub Genes. A) The PPI network constructed. Hub genes are highlighted as pink in the figure. B) The list of top 10 hub genes identified. C) ROC curves for the identified hub genes. The area under the curve (AUC) values for these hub genes were assessed to be greater than 0.8, indicating their strong diagnostic potential.

The Epi-DEs were used to construct a PPI network with the STRING database. Subsequently, using the CytoHubba application, the highly connected hub genes were identified respectively. The identified top 10 hub genes identified that are likely central to the constructed PPI network were *RBBP7*, *HDAC2*, *EZH2*, *SMARCA5*, *SUV39H1*, *CTCF*, *DNMT1*, *MORF4L1*, *ARID4B* and *KDM5B* respectively. ROC analysis evaluated the accuracy of the selected hub genes in predicting diagnostic status. The expression levels of the top 10 hub genes showed significant diagnostic value, as indicated by their area under the ROC curve (AUC) values (Fig. 3).

**Figure 4:**
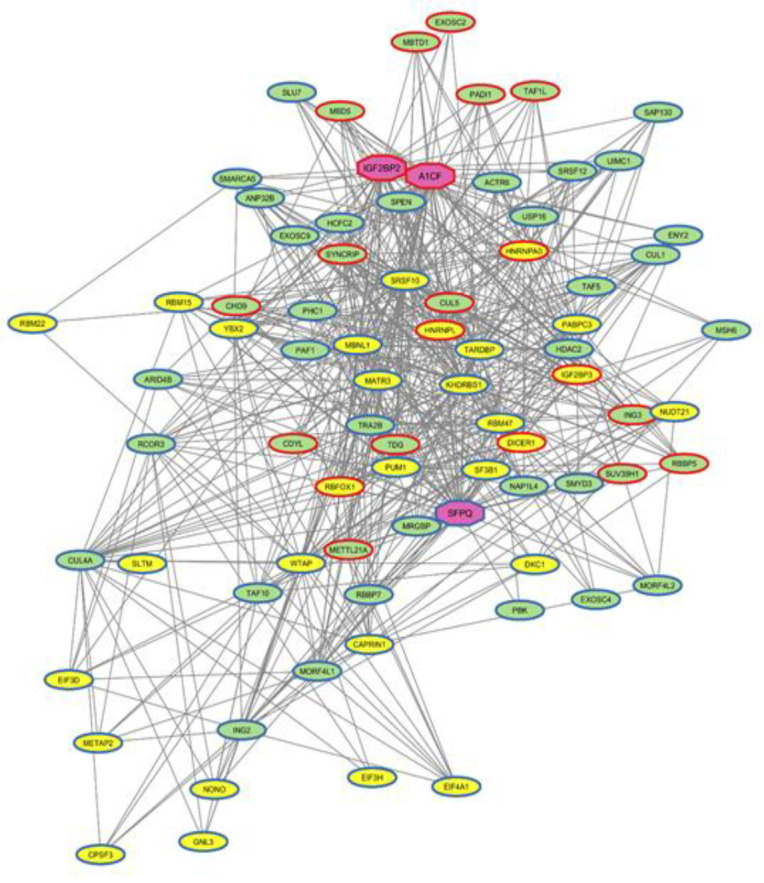
RPI networks constructed.

Given the critical role of RNA-binding proteins (RBPs) in testis development and spermatogenesis, this type of proteins were selected for investigation during RPI analysis [26]. The RPIs for the 63 Epi-DEGs were retrieved from the RNAInter database. In this interaction network, for 46 Epi-DEGs, 32 RNA-binding proteins (RBPs) were identified (Fig. 4). The top three key master regulators in this network, based on the number of interactions, were IGF2BP2, SFPQ and A1CF (Table 2).

**Table 2:** Master RBP regulators list and their information

**Master Regulator**	**Condition**	**LogFc**	**TYPE**	**Number of Targets**
IGF2BP2	Up	2.617896908	RBP	50
SFPQ	Down	-1.46074	RBP	48
A1CF	Up	1.163899	RBP	45

To further explore the role of *IGF2BP2*, it was valuable to perform enrichment analysis by the Reactome Pathways 2024 database on the target genes of *IGF2BP2* in the regulatory network to shed light on their functional roles. The top 10 enriched pathways are shown in Table 3. Overall, the *IGF2BP2* had 50 targets in the network in which, and out of all the hub genes, only the gene *SUV39H1* was not part of these protein targets (Table S6). These results can highlight the central role of *IGF2BP2* in the network constructed.

Epi-DEGs are represented in green. Yellow color spectrum circles represent RBP. The pink hexagon represents RBP master regulators. We have marked the upregulated genes with a red outline and the downregulated genes with a blue outline around them.

Given the essential role of RNA-binding proteins (RBPs) in gametogenesis and their contribution to the regulation of mRNA translation and decay during spermatogenesis, *IGF2BP2*, exhibiting the most significant change in expression among the RBPs, was chosen as a candidate for siRNA design [27]. 

**Table 3:** Pathway Enrichment Analysis for *IGF2BP2* and its target genes in PPI

**NO**	**Name**	**Adjusted ** ***p*** **-value**
**1**	Chromatin Modifying Enzymes	3.339e-12
**2**	Chromatin Organization	3.339e-12
**3**	Epigenetic Regulation of Gene Expression	2.778e-7
**4**	HATs Acetylate Histones	7.931e-7
**5**	Gene Expression (Transcription)	0.000002477
**6**	NoRC Negatively Regulates rRNA Expression	0.00007175
**7**	Negative Epigenetic Regulation of rRNA Expression	0.00007933
**8**	Post-translational Protein Modification	0.00007933
**9**	Regulation of PTEN Gene Transcription	0.0005918
**10**	PTK6 Regulates Proteins Involved in RNA Processing	0.001922

**Figure 5:**
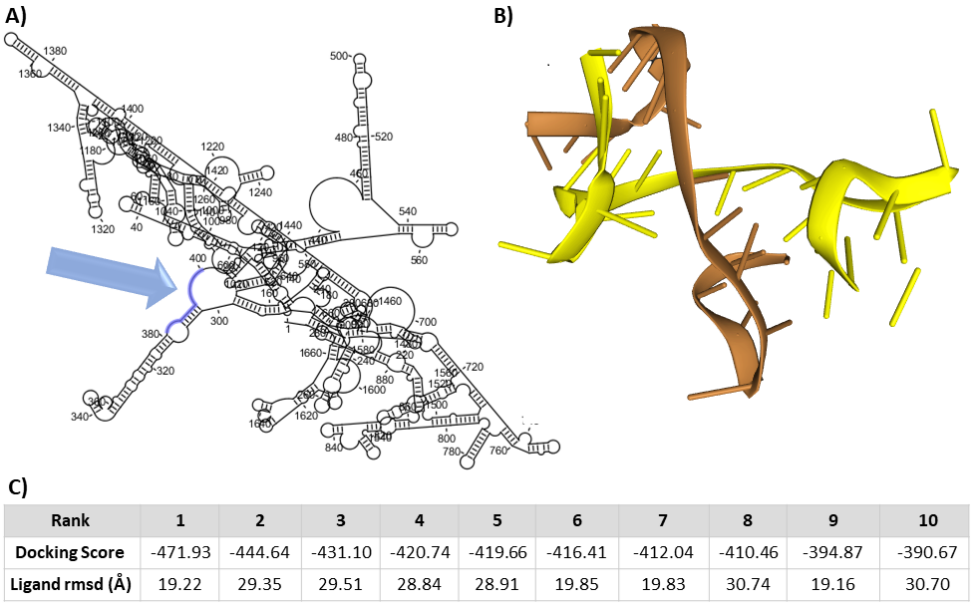
siRNA design.

The siRNAs against *IGF2BP2* **(**NM_001007225.3) were designed by Sfold a web server. The results of Sfold and IDT are mentioned in Table 4. The percentage of GC content in siRNA which is in the range of 40 to 60% is acceptable. Less or more than this amount will affect the structural stability of siRNA and prevent their proper functioning). siRNA binding energy is acceptable (more negative values ​​are better). Also, the investigations carried out with the Ensembl server showed that the best candidate designed siRNA E-value for *IGF2BP2* mRNAs is 0.034 and does not interact significantly with other mRNAs in the database of this server. Interaction between the best candidate siRNA and its target site on the *IGF2BP2* mRNA, as generated by the HADDOCK server, was also acceptable (Fig. 5).

**Table 4:** Master regulators list and their information

**Target position **(start - end)	**Sense siRNA** (5’-3’)	**Antisense siRNA** (5’-3’)	**GC content**	**Antisense siRNA binding energy **(kcal/mol)	**Hairpin** **G** (kcal/mol)
339-357	AGUCAACACAGACACAGAATT	UUCUGUGUCUG UGUUGACUTT	42.1%	-13.1	-1.5
340-358	GUCAACACAGACACAGAAATT	UUUCUGUGUCU GUGUUGACTT	42.1%	-14.4	-1.5
381-400	GCAACAAGAGAAGAAGCAATT	UUGCUUCUUCU CUUGUUGCTT	42.1%	-12.3	-1.5

## DISCUSSION

During male gamete differentiation, the genome undergoes major changes that affect the nuclear structure and epigenetic information [28]. Epigenetic processes play a crucial role in the regulation of gene expression. Another layer of dynamic gene regulation has been added lately with the finding that mRNA experiences comparable chemical changes that have a significant influence on translation and transcript turnover [29]. The sperm epigenome is highly variable and fluctuates over time. Consequently, spermatogenesis is particularly vulnerable to epigenetic alterations, which can result in spermatogenic abnormalities and infertility [30]. 

Spermatogenesis is a complex process involving over 2,000 different genes. Animal models have demonstrated that the knockout of numerous genes such as epigenetic regulators leads to subfertility [31]. Several studies have highlighted the importance of epigenetic regulators, including *ARID4B*, *EZH2*, *CUL4*, and *KHDRBS3*, in spermatogenesis and their association with male infertility [32-43]. 

A) The siRNA target site (blue arrow) on the *IGF2BP2* mRNA secondary structure is predicted using the CLC Genomics Workbench software. B) Interaction of the best siRNA with its binding site on the *IGF2BP2* mRNA as predicted by the HNADOCK server C) Docking Scores of the 10 models generated by the HNADOCK server for the interaction between the best siRNA and its target site on the *IGF2BP2* mRNA.

This study performed a gene expression profiling to identify epigenetic regulators with potential roles in teratozoospermia. Several key epigenetic regulators were found, some of which were previously reported their roles in male infertility and spermatogenesis, while others warrant further research. For instance, one study explored the role of IGF2BP2 in male infertility by examining its regulation of *PLOD2* mRNA stability. It was found that IGF2BP2 binds to the m6A-modified 3′UTR region of *PLOD2* mRNA, enhancing its stability. This increased stability leads to the upregulation of *PLOD2 *expression, which plays a significant role in regulating the proliferation and apoptosis of GC-2 cells (mouse germ cells) under oxidative stress conditions induced by varicocele [44]. Another study validated the expression of *PSPC1*, *NONO*, and *SFPQ* proteins in Sertoli cells within adult mouse testis sections. These findings indicate that PSPC1, NONO, and SFPQ likely interact to form complexes in Sertoli cells and could play a role in modulating transcriptional activity mediated by the androgen receptor [45]. Partial reduction in the levels of A1CF, an RNA-binding cofactor involved in APOBEC1-mediated RNA editing, or Argonaute 2 (AGO2), a crucial component in the biogenesis of specific noncoding RNAs, influences the risk of testicular abnormalities. A1CF plays a significant role in sustaining long-term reproductive performance. These results highlight the direct involvement of the RNA-binding proteins A1CF and AGO2 in the epigenetic regulation of germ-cell fate, urogenital development, and gamete functionality [46]. 

Our study highlights the crucial role of epigenetic-related genes in male gametogenesis and teratozoospermia. By integrating and analyzing microarray data, several key epigenetic regulators—including DNA methyltransferases, chromatin remodeling proteins, RNA-binding proteins, and enzymes involved in histone and DNA modifications—were identified. Additionally, our findings emphasize the potential of RBPs as diagnostic biomarkers and therapeutic targets for further investigation. Further research is needed to fully delineate the epigenetic pathways controlling normal spermatogenesis and identify additional molecular targets for identifying the reasons for teratozoospermia. Developing a comprehensive picture of the sperm epigenomic changes in teratozoospermia will be crucial for advancing diagnostic and therapeutic strategies for male infertility. Overall, this study provides valuable insights into the epigenetic underpinnings of teratozoospermia.
